# Identification and Characterization of a Novel *CLCN7* Variant Associated with Osteopetrosis

**DOI:** 10.3390/genes11111242

**Published:** 2020-10-22

**Authors:** Dmitrii S. Bug, Ildar M. Barkhatov, Yana V. Gudozhnikova, Artem V. Tishkov, Igor B. Zhulin, Natalia V. Petukhova

**Affiliations:** 1 Bioinformatics Research Center, Pavlov First Saint Petersburg Medical State University, St. Petersburg 197022, Russia; bug.dmitrij@gmail.com (D.S.B.); artem.tishkov@gmail.com (A.V.T.); 2 R.M. Gorbacheva Scientific Research Institute of Pediatric Hematology and Transplantation, Pavlov First Saint Petersburg State Medical University, St. Petersburg 197022, Russia; i.barkhatov@gmail.com; 3 Pavlov First Saint Petersburg State Medical University Clinic, St. Petersburg 197022, Russia; y.gudozhnikova@mail.ru; 4 Department of Microbiology and Translational Data Analytics Institute, The Ohio State University, Columbus, OH 43210, USA

**Keywords:** genetics, comparative genomics, phylogenetic analysis, osteopetrosis, *CLCN7* gene

## Abstract

Osteopetrosis is a group of rare inheritable disorders of the skeleton characterized by increased bone density. The disease is remarkably heterogeneous in clinical presentation and often misdiagnosed. Therefore, genetic testing and molecular pathogenicity analysis are essential for precise diagnosis and new targets for preventive pharmacotherapy. Mutations in the *CLCN7* gene give rise to the complete spectrum of osteopetrosis phenotypes and are responsible for about 75% of cases of autosomal dominant osteopetrosis. In this study, we report the identification of a novel variant in the *CLCN7* gene in a patient diagnosed with osteopetrosis and provide evidence for its significance (likely deleterious) based on extensive comparative genomics, protein sequence and structure analysis. A set of automated bioinformatics tools used to predict consequences of this variant identified it as deleterious or pathogenic. Structure analysis revealed that the variant is located at the same “hot spot” as the most common *CLCN7* mutations causing osteopetrosis. Deep phylogenetic reconstruction showed that not only Leu614Arg, but any non-aliphatic substitutions in this position are evolutionarily intolerant, further supporting the deleterious nature of the variant. The present study provides further evidence that reconstructing a precise evolutionary history of a gene helps in predicting phenotypical consequences of variants of uncertain significance.

## 1. Introduction

Osteopetrosis (“marble bone disease”) is a group of rare inheritable disorders of the skeleton characterized by increased bone density. The estimated prevalence of osteopetrosis is 1 in 100,000 to 500,000 [1,2]. It exists in three clinical forms based on the pattern of inheritance: a benign autosomal dominant form (ADO), a malignant autosomal recessive form (ARO) with incidents 1 in 20,000 and 1 in 250,000 births, respectively, and the X-linked form. The autosomal dominant adult (benign) form is associated with few, if any, symptoms, and the autosomal recessive infantile (malignant) form is typically fatal during infancy or early childhood if untreated [1,3]. General skeletal abnormality can be characterized by increased bone density, diffuse and focal sclerosis, and pathological fractures. Osteopetrosis comprises a clinically and genetically heterogeneous group of conditions that share the hallmark of increased bone density on radiographs. The increase in bone density results from abnormalities in osteoclast differentiation or function. The Nosology Group of the International Skeletal Dysplasia Society classifies increased bone density conditions into several distinct entities based on clinical features, mode of inheritance and underlying molecular and pathogenetic mechanisms [4]. Treatment of osteopetrosis is mostly symptomatic, with some exceptional cases of transplanting hematopoietic stem cells. However, the disease is remarkably heterogeneous in clinical presentation and often misdiagnosed. Therefore, genetic testing and molecular pathogenicity analysis are essential for precise diagnosis and new targets for preventive pharmacotherapy [5].

Osteopetrosis is caused by the failure of osteoclast differentiation or function and mutations in at least 10 genes have been identified as causative in humans and collectively account for approximately 80% of patients in the cohorts [5,6]. The genetic basis of this disease is now largely uncovered: mutations in *TCIRG1*, *CLCN7*, *OSTM1*, *SNX10* and *PLEKHM1* lead to osteoclast-rich ARO (in which osteoclasts are abundant but have severely impaired resorptive function), whereas mutations in *TNFSF11* and *TNFRSF11A* lead to osteoclast-poor ARO. In osteoclast-rich ARO, impaired endosomal and lysosomal vesicle trafficking results in defective osteoclast ruffled-border formation and, hence, the inability to resorb bone and mineralized cartilage [2]. Mutations in *TCIRG1* and *CLCN7* together account for nearly 70% of all patients with ARO. Mutations in the *CLCN7* gene are responsible for about 75% of cases of ADO, 10–15% of cases of autosomal recessive osteopetrosis, and all known cases of intermediate autosomal osteopetrosis. Mutations in the *CLCN7* gene affect the function of osteoclast-mediated extracellular acidification, resulting in the disturbed dissolution of the bone inorganic matrix and a series of clinical features [7].

The chloride channel 7 (*CLCN7*) gene is a member of the mammalian CLC gene family. In osteoclasts, the CLCN7 protein resides in the late endocytotic–lysosomal pathway of the ruffled membrane borders and is involved in the acidification of the resorption lacunae [8]. The physiological function of the CLC7 protein was unclear until it was shown that the disruption of the *CLCN7* gene in mice causes severe osteopetrosis, and that the CLC7 protein played an essential role in the acidification of the extracellular resorption lacunae, which is important for osteoclast-mediated resorption of mineralized bone. In summary, *CLCN7* is essential for bone remodeling, and mutations of this gene lead to deviant, brittle osseous structure. Bone condensation results in an abnormal structure of the osseous matter, making the bone brittle. This leads to an increased risk of fractures that most frequently involve the upper one-third of the femur and the tibia [9]. In humans, mutations in the *CLCN7* gene give rise to the complete spectrum of osteopetrosis phenotypes [10]. Here, we describe a newly identified variant of the *CLCN7* gene in a patient diagnosed with osteopetrosis and provide evidence that this novel missense mutation is deleterious.

## 2. Materials and Methods

### 2.1. Case Report

A 14-year-old male patient was diagnosed with osteopetrosis in the Pavlov First St. Petersburg Medical State University Clinic. The patient’s legal guardian provided written informed consent for genomic sequencing and research. The study was conducted in accordance with the Declaration of Helsinki, all aspects were reviewed and approved by the Ethics committee at the Pavlov First St. Petersburg State Medical University, Russian Federation (protocol 35-2020). The patient’s legal guardian provided written informed consent for publication of this case report and any accompanying images.

### 2.2. DNA Sequencing

Patient’s whole exome sequencing (WES) was performed on Illumina NextSeq500 by iBinom (Moscow, Russia). Human genome 19 (hg19) build 37 was used as a reference sequence. The following genes associated with osteopetrosis were screened for variants: *TCIRG1*, *CLCN7*, *OSTM1*, *RANKL*, *RANK*, *IKBKG*, *SNX10*, *TNFSFR11A*, *TNFSF11*, *PLEKHM1*, *CA2*. This approach identified a previously unknown heterozygous variant in the *CLCN7* gene. No other variants were uncovered that might account for the disease phenotype. The variant was submitted to ClinVar (https://www.ncbi.nlm.nih.gov/clinvar/) (accession: SCV001190007). Raw sequencing data were deposited in BioProject database (https://www.ncbi.nlm.nih.gov/bioproject/) (SRA (Sequence Read Archive) accession: PRJNA613088). Sanger sequencing was performed to verify this variant. Polymerase chain reaction (PCR) products of the gene locus were obtained by protocol suggested for 20 exone [11] and subjected to sequencing by using BigDye^®^ Terminator v3.1 cycle sequencing kit on an AB3500xl genetic analyzer (Applied Biosystems, Waltham, MA, USA).

### 2.3. Sequence Acquisition

Reference sequences of the *CLCN7* gene (NG_007567.1), coding nucleotides (NM_001287.6) and amino acids (NP_001278.1) were retrieved from the NCBI database.

### 2.4. Bioinformatics Analysis

CLC7 protein sequence domain architecture was analyzed using CDvist (http://cdvist.zhulinlab.org/) [12]. A 3D homology model of the CLCN7 protein was built by the Swiss-Model server (https://swissmodel.expasy.org/) [13] using the structure of a eukaryotic CLC protein [14] as a template (PDB accession: 3ORG). BLAST searches of the NCBI RefSeq database were carried out with default parameters using CLCN7 protein sequence (NP_001278.1) as a query. Multiple sequence alignments were constructed using MUSCLE 3.8.31 [15] and edited in JalView 2.11.1.2 [16]. Neighbor-joining and maximum likelihood phylogenetic trees were built using MEGA 7.0 [17].

## 3. Results

### 3.1. Case Representation

**Figure 1:**
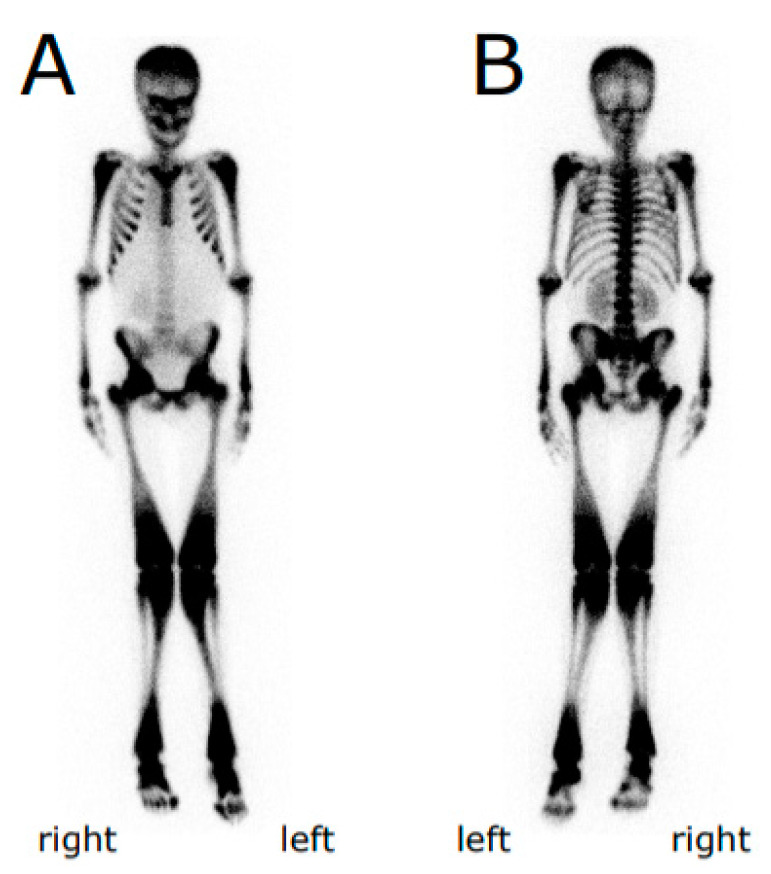
Bone scintigraphy of the proband. Anterior (**A**) and posterior (**B**) views. Metaphyses and diaphyses of humerus, femur, tibia and fibula are widened.

A 14-year-old male patient was diagnosed with osteopetrosis (anamnestic) based on the following symptoms: multiple (more than 10) bone fractures including right humerus fracture at 9 months of age and subperiosteal fracture of the left femur neck, signs of osteosclerosis, bone marrow failure, hepatosplenomegaly, and congenital anomalies (Figure 1, Table 1, Supplemental Table S1).

The patient had a 23-year-old sibling, who was also diagnosed with osteopetrosis based on clinical evaluation, patient history and X-ray imaging. Onset in late childhood/adolescence in both siblings suggested Type II ADO.

### 3.2. Mutation Analysis

**Figure 2:**
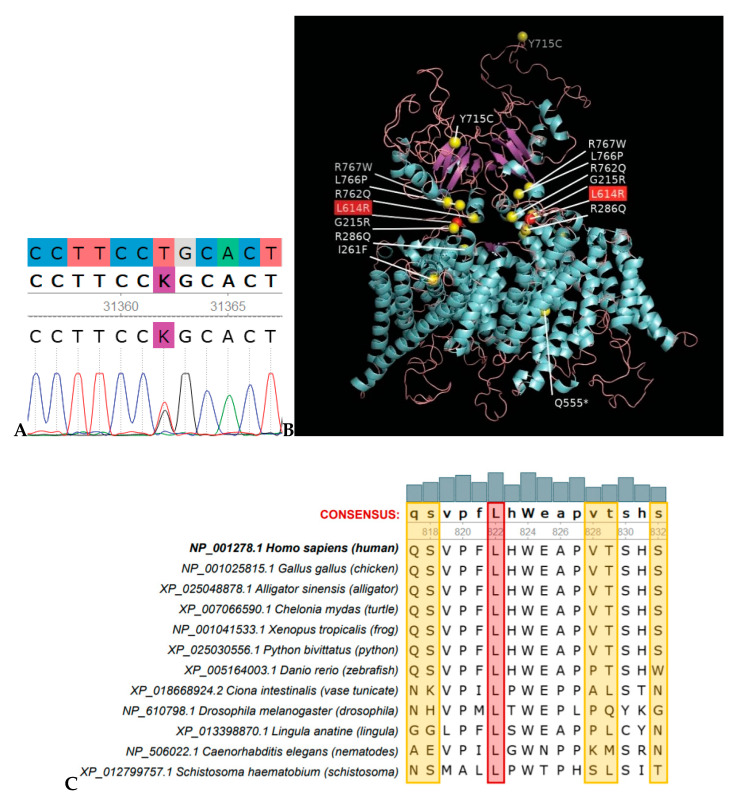
Leu614Arg substitution occurred in a mutational “hot spot” and it is evolutionarily intolerable. (**A**) Sanger sequencing showing the heterozygous c.1769T>G, p.Leu614Arg variant. (**B**) CLCN7 structural homology model by Swiss-Model (CmCLC, PDB accession 3ORG, was used as a template). The most common pathogenic variants causing osteopetrosis are shown as yellow spheres; the Leu614Arg variant is shown as a red sphere. (**C**) A fragment of multiple sequence alignment of CLCN7 orthologs from representative metazoan genomes. Position corresponding to Leu614 in the human CLCN7 protein is highlighted in red. Variable positions highlighted in yellow show that there was a significant time for divergence.

Screening of 11 genes associated with osteopetrosis (sequences obtained by low-coverage WES) identified a previously unknown heterozygous variant in the *CLCN7* gene (Table 2): NM_001287.6:c.1841T>G (NP_001278.1:p.Leu614Arg), consistent with the fact that mutations in the *CLCN7* gene are responsible for about 75% of cases of Type II ADO [8]. The variant was confirmed by Sanger sequencing (Figure 2A). The c.T1769G (p.Leu614Arg) is novel and has not been reported in the 1000 Genomes Project (2504 samples, accessed 9/18/2019), dbSNP (accessed 03/20/2020), or the Genome Aggregation Database (gnomAD v.2.1.1, 125,748 exomes, and gnomAD v3, 71,702 genomes, accessed 03-20-2020). A different variant in the same position, Leu614Pro, was reported in the ClinVar database (rs1064794323, reported as “uncertain significance”) in a child with severe osteopetrosis, anemia, blindness, neurological impairment and macrocephaly, who also had a deletion in exon 17 of the *CLCN7* gene [12]. Another variant in the same position, Leu614Met, was reported in the dbSNP database (rs1000353389).

### 3.3. Comparative Protein Sequence Analysis and Modelling

CLCN7 protein domain analysis using CDvist revealed 11 transmembrane helices, N- and C-terminal low complexity regions, and two C-terminal CBS-domains (Supplemental Figure S1). We built a 3D homology model of the CLCN7 protein using the Swiss-Model server and the structure of a eukaryotic CLC protein [14] as a template (PDB accession: 3ORG). Position Leu614 is located at the C-terminal low complexity region between the last transmembrane helix and the first CBS (cystathionine β-synthase) domain (Supplemental Figure S1). Notably, this position is found at the same location in the protein tertiary structure as the most common mutations causing osteopetrosis—almost all of them are clustered at the intracellular gates of the CLC channel dimer, the CLCN7 mutational “hot spot” (Figure 2B).

### 3.4. Bioinformatics Analysis for Variant Significance Confirmation

We used several bioinformatics tools, all of which predicted that the newly identified Leu614Arg variant is damaging (Supplemental Table S2). However, strictly following American College of Medical Genetics (ACMG) guidelines for interpretation [18], the CLCN7 p.Leu617Arg variant should be classified as “variant of uncertain significance” based on two moderate and one supporting criteria. Therefore, in addition to automated bioinformatics tools, we used a recently developed evolutionary approach based on removing paralogous sequences from analysis [19]. We identified eight paralogs of CLCN7 in the human genome (Supplemental Figure S2). In order to exclude paralogs from further analysis, we searched for orthologs of nine human proteins (CLCN7 and its paralogs) among the representative Metazoan genomes (Supplemental Figure S3). Species taken in the analysis were evenly distributed over the Tree of Life, with several species per class and approximately one species per order. Then BLAST search for CLCN7 against the NCBI RefSeq database was performed against the representative genomes. From the resulting sequences, a neighbor-joining phylogenetic tree was constructed to reveal CLCN7 orthologs, and exclude all paralogs. Multiple sequence alignment of CLCN7 orthologs showed 100% conservation of Leu614 (Figure 2C).

In addition, the human CLCN7 protein sequence was used as a query in a BLAST search against all Metazoan genomes. The first 1000 resulting sequences were aligned and a neighbor-joining phylogenetic tree was constructed. Using orthologous/paralogous relationships defined with the set of representative genomes, we were able to identify a clade of CLCN7 orthologs, a clade with CLCN6 orthologs and mixed (CLCN6 and CLCN7) wedge-like clade containing only representatives of invertebrates (Supplemental Figure S4). In the resulting alignment, only four amino acid residues were found in a position corresponding to L614 in the human CLCN7: Leu = 97.0% (970), Met = 1.3% (13), Ile = 0.3% (3), Val = 0.3% (3). These results show that only aliphatic amino acids and, functionally related to them, methionine, are allowed in this position even among close paralogs. A variant Leu614Met reported in dbSNP (rs1000353389) can therefore be considered evolutionarily permitted. In contrast, a substitution Leu614Arg is evolutionarily intolerable and thus should be classified as damaging.

## 4. Discussion

In this study, we report the identification of a novel variant in the *CLCN7* gene in a patient diagnosed with osteopetrosis and provide evidence for its significance. Although several independent lines of indirect evidence suggested that the newly detected CLCN7 p.Leu617Arg variant is damaging, according to ACMG guidelines for interpretation [18] it should be classified as a “variant of uncertain significance”. Just one supporting criterion was needed to re-classify it to “likely damaging”; however, because mutations in the *CLCN7* gene account for 75%, not 100% cases, this criterion was not met. On the other hand, we provide a strong argument for this being a likely pathogenic variant by revealing the precise evolutionary history of this gene and showing that all changes to non-aliphatic amino acids in position Leu617 were selected against during hundreds of millions of years. Not a single case of successful substitution to non-aliphatic amino acid in this position can be found among hundreds of CLCN7 orthologs. It is important to stress that this is not a computational prediction, but a direct observation: mutation Leu617Arg is evolutionarily intolerable and, therefore, damaging. Taken together with the fact that this variant is found in a gene, mutations in which account for the vast majority of autosomal dominant osteopetrosis cases, and it is located in a mutational “hot spot” of this gene, our evolutionary analysis strongly suggests that this variant is deleterious and, therefore, likely pathogenic.

In conclusion, we identified a novel, deleterious variant in the *CLCN7* gene. Genomic and protein structure analyses suggest that this variant is located in a mutational “hot spot” and it is evolutionarily intolerable.

## Figures and Tables

**Table 1:** Phenotypic features of the proband associated with osteopetrosis autosomal dominant form (ADO) type 2.

Autosomal Dominant Osteopetrosis Type 2 Clinical Features	Proband	Sibling	Relevance/Alternate Explanation
Autosomal dominant inheritance	Yes	Unknown	
Facial nerve palsy	No	Unknown	
Vision loss, severe, beginning in childhood	No	Unknown	
Osteosclerosis, diffuse symmetrical	Yes	Unknown	
Increased long bone fracture rate (75% of patients)	Yes	Yes	
Multiple fractures	Yes	Yes	
Pronounced skull base sclerosis	Unknown	Unknown	
Mandibular osteomyelitis	No	Unknown	
‘Rugger-Jersey’ spine (vertebral endplate thickening)	Unknown	Unknown	
Endobones (bone within bone)	Unknown	Unknown	
Hip osteoarthritis	No	Unknown	
Facial palsy due to cranial nerve VII compression	No	Unknown	
Bone marrow failure	Yes	No	
Elevated serum acid phosphatase	Unknown	Unknown	
Onset in childhood	Yes	Yes	
Progressive sclerosis with age	Unknown	Unknown	
20–40% patients are asymptomatic	No	No	
**Other Clinical Features**			
Hepatomegaly	Yes	Unknown	Clinical feature of autosomal recessive osteopetrosis type 4 (OMIM* #611490)
Splenomegaly	Yes	Unknown	Same as above
Anemia	Yes	Unknown	Same as above
Reticulocytosis	Yes	Unknown	Same as above
Thrombocytopenia	Yes	Unknown	Same as above
Failure to thrive	Yes	Unknown	Clinical feature of autosomal recessive osteopetrosis type 1 (OMIM #259700)
Hydrocephalus	Yes	Unknown	Same as above
Splayed metaphyses	Yes	Unknown	Same as above
Low serum calcium	Yes	Unknown	Same as above
Elevated alkaline phosphatase	Yes	Unknown	Same as above
Valgus deformity	Yes	Unknown	Clinical feature of autosomal recessive osteopetrosis type 2 (OMIM #259710)
Dental anomalies	Yes	Unknown	Same as above
Elevated serum lactate dehydrogenase	Yes	Unknown	Clinical feature of autosomal recessive osteopetrosis type 5 (OMIM #259720)
Lymphocytosis	Yes	Unknown	Assumed related
Skeletal effects	Yes	Unknown	Assumed related
Scoliosis	Yes	Unknown	Assumed related
Low hairline	Yes	Unknown	Assumed related
Double xiphoid process	Yes	Unknown	Assumed related

OMIM*: Online Mendelian Inheritance in Men (an online catalogue of human genes and genetic disorders).

**Table 2:** Genomic findings newly identified in the proband diagnosed with osteopetrosis.

Gene	Genomic Location	DNA Reference	Protein Reference	Variant Type	Genotype	Origin	Observed Effect
*CLCN7*	Chr16: 1498724 (GRCh37)	NM_001287.6: c.1841T>G	NP_001278.1: p.Leu614Arg	missense	heterozygous	Unknown	Deleterious

## Supplementary Materials

The following are available online at https://www.mdpi.com/2073-4425/11/11/1242/s1, Figure S1: Predicted domain architecture of the CLCN7 protein, Figure S2: Neighbor-joining phylogenetic tree of CLCN7 paralogs identified in the human genome, Figure S3: Neighbor-joining phylogenetic tree of CLCN7 orthologs from representative metazoan genomes, Figure S4: Separation of CLCN7 orthologs from the closely related paralogs. Table S1: Clinical features of the proband, Table S2: Functional effect of mutation L614R by different SNP predictors.
