# Supplementary material for: Identification and Characterization of a Novel CLCN7 Variant Associated with Osteopetrosis
Source: Genes (Basel). 2020 Oct 22;11(11):1242. doi: 10.3390/genes11111242 (PMC7690398; doi:10.3390/genes11111242)
Supplement: Supplementary file 1 [file genes-11-01242-s001.pdf]

Supplementary Material

Identification and characterization of a novel *CLCN7* variant associated with osteopetrosis

Dmitrii S. Bug[1], Ildar M. Barkhatov[2], Yana V. Gudozhnikova[3], Artem V. Tishkov[1], Igor B. Zhulin[1,4*] and Natalia V. Petukhova[1*]

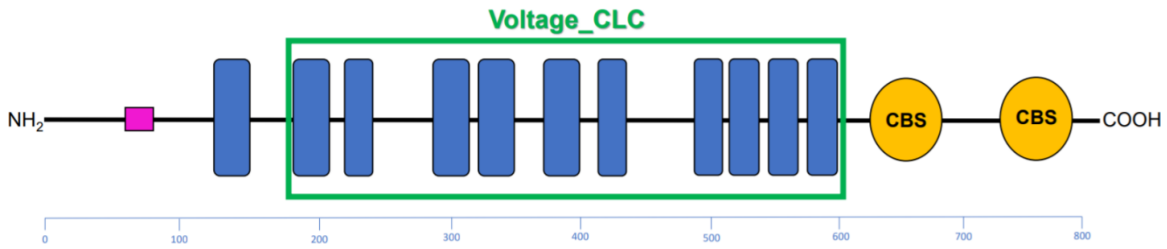

Supplemental Figure 1: Predicted domain architecture. Low complexity region colored in pink; transmembrane regions are indicated by blue boxes; CBS - cystathionine beta synthase domain; green box shows Voltage-gated chloride channels superfamily.

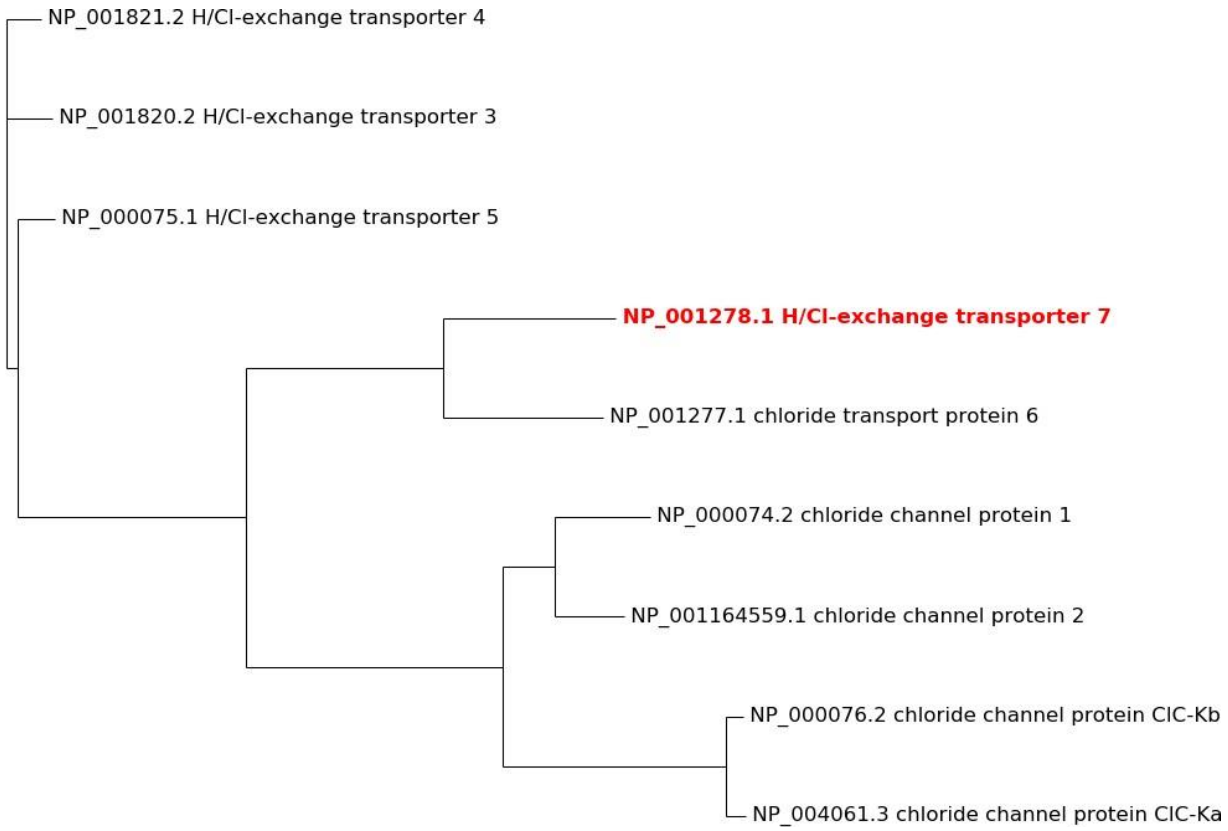

Supplemental Figure 2: Neighbor-joining phylogenetic tree of CLCN7 paralogs identified in the human genome. The product of *CLCN7* gene is shown in red.

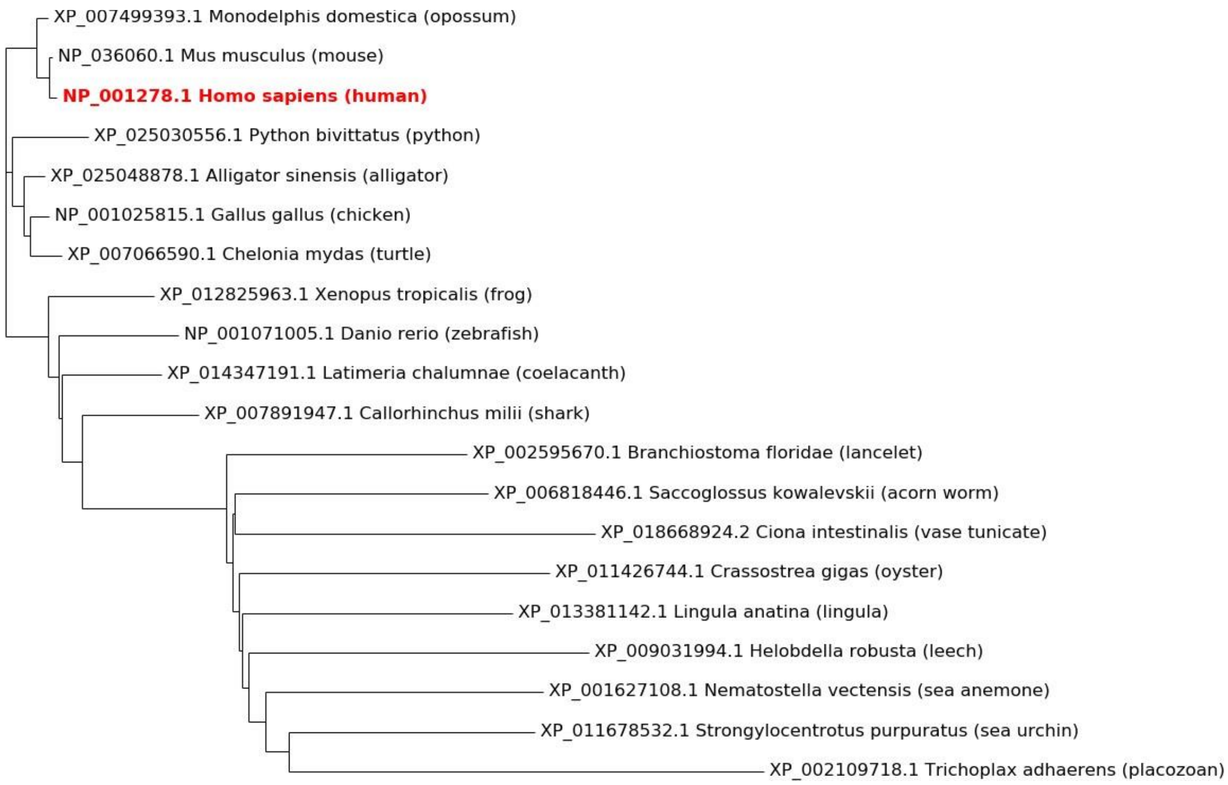

Supplemental Figure 3: Neighbor-joining phylogenetic tree of CLCN7 orthologs from representative metazoan genomes.

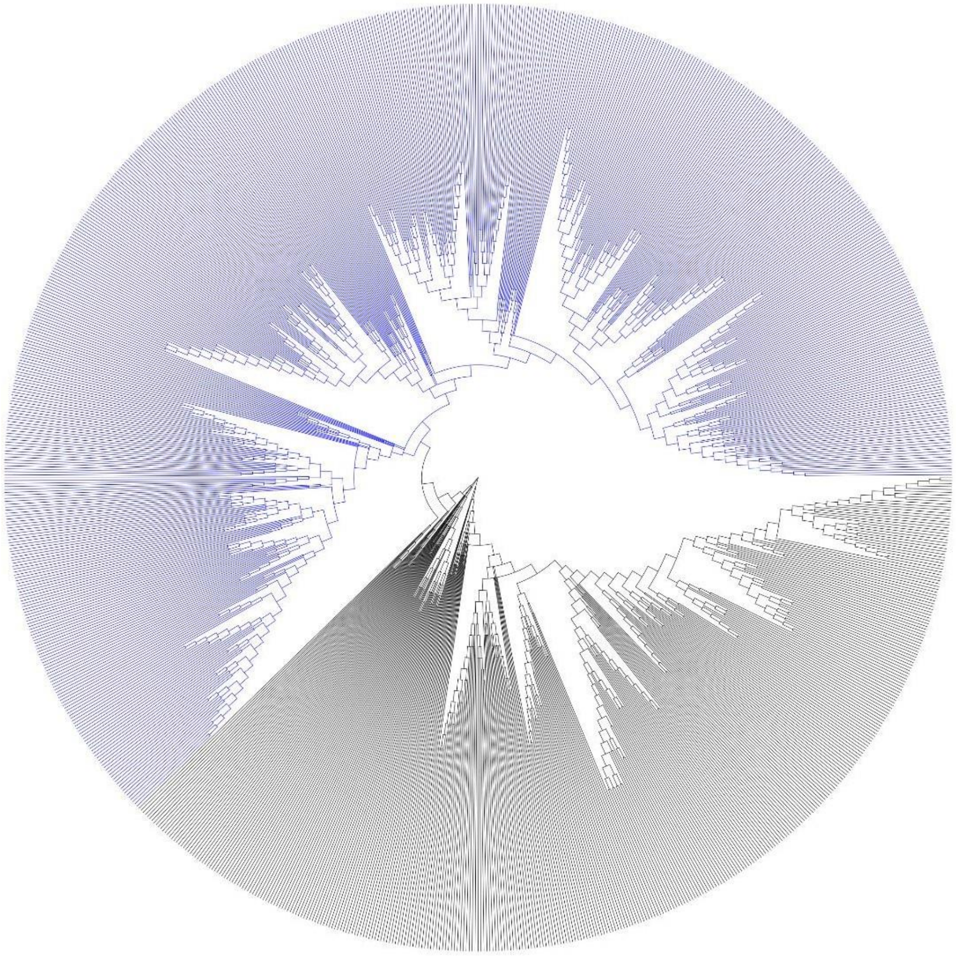

Supplemental Figure 4: Separation of CLCN7 orthologs from the closely related paralogs. The neighbor-joining phylogenetic tree contains 1000 CLCN6 and CLCN7 protein sequences from the RefSeq database (limited to Metazoa). The CLCN7 clade (shown in blue) is separated from the rest of the tree by the longest branch.

Supplemental Table 1: Clinical features of the proband.

Clinical Features	Comment
Osteosclerosis, diffuse symmetrical	Bone density studies showed increasing of Z-score in lumbar vertebrae from 9.2 to 10.6, as well as symmetrical widening of diaphyses and metaphyses of femur, fibula, tibia and humerus.
Increased long bone fracture rate (75% of patients)	Not observed.
Multiple fractures	More than ten.
Bone marrow failure	Bone marrow biopsy showed erythroid hyperplasia (>50% of cells), and signs of diserythropoiesis: deformed erythroblasts nuclei, the presence of binucleated cells and megaloblasts.
Onset in childhood	Proband diagnosed at the age of four.
Hepatomegaly	Maximum liver dimension is 16.6.
Splenomegaly	Enlarged spleen occupies half of the abdominal cavity. Splenic vein diameter increased up to 1.4 cm.
Anemia	Hemoglobin level decreased to 46 g/l.
Reticulocytosis	Reticulocyte count is 9.83 %.
Thrombocytopenia	Platelet count is 17 109/l.
Failure to thrive	Proband weighed 45 kg.
Hydrocephalus	Not observed.
Splayed metaphyses	Bone scintigraphy showed splayed metaphyses
Low serum calcium	2.16 mmol/l.
Elevated serum alkaline phosphatase	161.6 U/l.
Valgus deformity	Not observed.
Dental anomalies	Malocclusion.
Elevated serum lactate	631.6 U/l.

dehydrogenase	
Lymphocytosis	Lymphocyte count is 5.6 109/l.
Skeletal defects	Right leg is shortened by 2 cm.
Right thoracolumbar scoliosis	Not observed.
Low hairline	Not observed.
Double xiphoid process	Not observed.

Supplemental Table 2: Functional effect of mutation L614R by different SNP predictors.

**SNP predictor**	**Functional effect**	**Prediction score**
PolyPhen2	Probably damaging	1.000
SIFT	Affect protein function (probably damaging)	0.00
PROVEAN	Deleterious	-5.794
PANTHER	Disease causing variant	0.845
PhD-NP	Disease causing variant	0.910
Meta-SNP	Disease causing variant	0.850
PredictSNP	Deleterious	0.79
SNAP	Affect protein function (deleterious)	69
